# P54 Enhancing antibiotic efficacy through nanotechnology: innovations in targeted antimicrobial delivery

**DOI:** 10.1093/jacamr/dlaf118.061

**Published:** 2025-07-14

**Authors:** Aser Waleed Mohamed Marzok, Tala Ishola, Rasha Abdelsalam Elshenawy

**Affiliations:** School of Health, Medicine and Life Sciences, University of Hertfordshire, Hatfield, UK; School of Health, Medicine and Life Sciences, University of Hertfordshire, Hatfield, UK; School of Health, Medicine and Life Sciences, University of Hertfordshire, Hatfield, UK

## Abstract

**Background:**

Antibiotic resistance poses a growing global health threat, demanding innovative solutions to enhance treatment efficacy.^1^ Nanotechnology offers promising advancements by enabling precise antibiotic delivery, improving drug stability, and overcoming bacterial resistance mechanisms.^2^ By utilizing nanoparticles such as polymeric micelles, biomimetic structures, and inorganic materials, researchers aim to optimize antibiotic therapies.^3^ This study reviews recent developments in nanoparticle-mediated antibiotic delivery, highlighting key trends and future directions for clinical application.

**Objectives:**

This study aims to explore how nanotechnology can improve antibiotic efficacy by more precisely targeting bacterial infections, reducing systemic side effects, and overcoming antimicrobial resistance.

**Methods:**

A systematic literature review was conducted across PubMed, SCOPUS, Google Scholar, and Web of Science, covering studies published between 2020 and 2025. Peer-reviewed articles focusing on nanoparticle-based antibiotic delivery systems were included, while studies lacking transparent methodology or clinical relevance were excluded. A thematic qualitative analysis was performed to identify key trends and emerging technologies in the use of nanoparticles to enhance antibiotic therapies.

**Results:**

Out of 1350 studies initially identified, 45 studies met the inclusion criteria and were subjected to detailed analysis. The findings highlighted that polymer-based micelles, biomimetic liposomes, and inorganic nanoparticles are pivotal in advancing antibiotic delivery strategies. These nanoparticle (NP) systems significantly improved antibiotic stability, enabled controlled and targeted drug release, enhanced bacterial targeting, and demonstrated superior therapeutic efficacy compared to conventional antibiotic formulations. Key pharmacokinetic advantages included enhanced solubility, absorption, distribution, and a notable reduction in systemic toxicity. Among the different systems, polymeric nanoparticles were the most extensively studied, showing remarkable potential in facilitating intracellular antibiotic uptake. Biomimetic nanoparticles, designed to mimic natural biological systems, enhanced drug delivery efficiency by improving biocompatibility and cellular interaction. Inorganic nanoparticles, notably silver and gold, were particularly effective in overcoming bacterial resistance by disrupting bacterial membranes and facilitating deeper antibiotic penetration.

**Conclusions:**

Nanotechnology offers a powerful platform for improving antibiotic therapies by enabling targeted drug delivery, reducing adverse effects, and addressing antimicrobial resistance. Thematic analysis identified three dominant trends: the extensive use of polymeric nanoparticles for drug delivery, the innovative application of biomimetic nanoparticles to enhance cellular uptake, and the utilization of inorganic nanoparticles to counteract bacterial resistance mechanisms. Despite these promising advancements, challenges remain, including ensuring consistent efficacy across diverse bacterial strains and preventing microbial adaptation to nanoparticle-based therapies. Future research should prioritize the development of optimized nanoparticle formulations, extensive clinical evaluations, and strategies for integrating nanoparticle technologies into mainstream clinical practice. Overall, nanotechnology holds great promise in revolutionizing antibiotic treatment and tackling the global threat of antimicrobial resistance.

## References

[1] Abdelsalam Elshenawy R, Umaru N et al WHO AWaRe classification for antibiotic stewardship: tackling antimicrobial resistance - a descriptive study from an English NHS Foundation Trust prior to and during the COVID-19 pandemic. Front Microbiol 2023; 14: 1298858. 38146447 10.3389/fmicb.2023.1298858 PMC10749484

[2] Parvin N, Joo SW, Mandal TK. Nanomaterial-based strategies to combat antibiotic resistance: mechanisms and applications. Antibiotics (Basel) 2025; 14: 207. 40001450 10.3390/antibiotics14020207 PMC11852044

[3] Wang Y, Sun H. Polymeric nanomaterials for efficient delivery of antimicrobial agents. Pharmaceutics 2021; 13: 2108. 34959388 10.3390/pharmaceutics13122108 PMC8709338

